# Enhancing the Role of the Nurse in Primary Care: The RN “Co-Visit” Model

**DOI:** 10.1007/s11606-015-3456-6

**Published:** 2015-07-21

**Authors:** Karen A. Funk, Malia Davis

**Affiliations:** Clinica Family Health, 1345 Plaza Court North, 1A, Lafayette, CO 80026 USA

**Keywords:** nursing, access to care, physician satisfaction, team-based care

*Every day, Dr. D felt exhausted by the end of a care session and wondered how much longer she could survive double-booked appointments, paperwork, and charting. While finishing her charting late each evening, she considered leaving.*

*J.C., one of the clinic’s RNs, spent all day phone-triaging patients who needed care now but had no access. She wondered if her nursing skills were being used and valued. She was tired of begging providers to double-book patients she felt should be seen. She left most days without her work done, and though she loved the patients, she wondered how long she could continue.*

With more and more Americans gaining access to primary care, clinics face the challenge of increasing demand for services with no enhanced capacity to meet the demand. The tension that arises in such a system is exacerbated by provider and nurse burnout and a recurring sense of under-providing healthcare. We addressed this issue by initiating RN-led visits that required little provider time, thereby increasing access without worsening burnout.

Clinica Family Health is a community health center with five clinical sites in communities northwest of downtown Denver, Colorado. We serve approximately 45,000 patients annually, with 98 % of our families living at or below 200 % of the federal poverty level. For 60 % of our patients, Spanish is the primary language, and virtually all of our providers and staff speak English and Spanish. We have a long history of innovation in care delivery, but by 2012, found ourselves struggling to sustain access with an eroding base of providers.^1^ We decided to restructure our care delivery model in order to maintain our success in achieving the triple aim, while increasing job satisfaction among care team members.

Our goal was to increase access by modestly expanding panel sizes and enhancing the team surrounding our providers. At the same time, we wanted to promote work/life balance for care team members and inspire loyalty in order to retain physicians, nurse practitioners, and physician assistants who were leaving for jobs with less pressure, more pay. We started with a literature review and analysis of our existing pod model: 14 care teams, or "pods," composed of medical doctors, nurse practitioners, physician assistants, nurses, medical assistants, a case manager, a behavioral health provider, and operations support staff. We evaluated each pod member’s role and scope. We completed time studies on 34 visits prior to implementation, focusing on patient experience. We also interviewed Clinica leaders and employees in other high-performing practices.

We learned through this process that we were not optimizing a hallmark of Clinica’s care team transformation—utilizing each team member to better serve patients while increasing joy in work. Of the 34 observed visits, only one involved a patient seeing a nurse. Our nurses’ primary role had become phone triage, not direct patient care. What’s more, a dearth of appointments had led to an uptick in triage calls. As one RN explained it, “Triaging with no access creates only more work, electronic tasks to finish, and extra phone calls… I hate telling patients to seek urgent care when I know we could help them here if we had space…”

Through financial analysis, we determined that increasing total daily visits per provider by appoximately 2 would achieve our access goal, while generating enough revenue to cover the cost of additional care team staff. Yet adding provider visits was in direct conflict with reducing provider burnout. Engaging our nurse colleagues in direct patient care was a solution to this dilemma.^2^

In 2014, we tested an RN “flip visit” or co-visit in two pods through a pilot program we called Pods 2.0. Co-visits address same-day acute complaints, and each nurse can perform up to eight co-visits a day. During a co-visit, the nurse elicits and records the history of present illness and past medical, family, and social histories. She collects vital signs, implements pertinent standing orders, and creates a nurse note. When the provider enters the room, the nurse presents the history to the provider in front of the patient, allowing the provider to clarify details. At this point, the nurse switches to a scribe role, documenting the provider’s physical exam, assessment, and plan. After the provider moves on to the next patient encounter, the nurse reviews the care plan and provides appropriate education with the patient. Later, the provider quickly reviews and authenticates the visit documentation before submission for billing, in accordance with Center for Medicaid and Medicare Services (CMS) guidelines.^3^

To staff the co-visits, we increased nursing full-time equivalents (FTEs) from 1.0 to 3.0 per pilot pod. We moved phone triage off the pod and piloted a triage-only nurse position. We eliminated provider double-booking. Using detailed guidelines, we layered in a separate nurse schedule for co-visits to be scheduled by a triage nurse or by our centralized communication center. We added an extra medical assistant to help with flow, medication refills and reconciliation, vaccine administration, and other tasks. One nurse on each care team engaged in traditional nursing care such as visits for patients taking warfarin, wound care, patient education, complex care management, and quality outcome auditing. The other two nurses performed the co-visits in conjunction with providers. The co-visit schedule ran in parallel with a provider schedule featuring a slot held for one to two co-visits with the nurse partner for every two standard visits completed by the provider (Fig. 1). Generally, flip visits took 20–30 min total, requiring about 10 min of provider time. RNs found any available provider on the care team to engage in co-visit care. Co-visits were used to provide greater access to patients and could also help fill holes created in the provider schedule by no-show patients. For the pilot, we “borrowed” staff from other care teams, with the unfortunate consequence of leaving other teams understaffed.

**Fig. 1:**
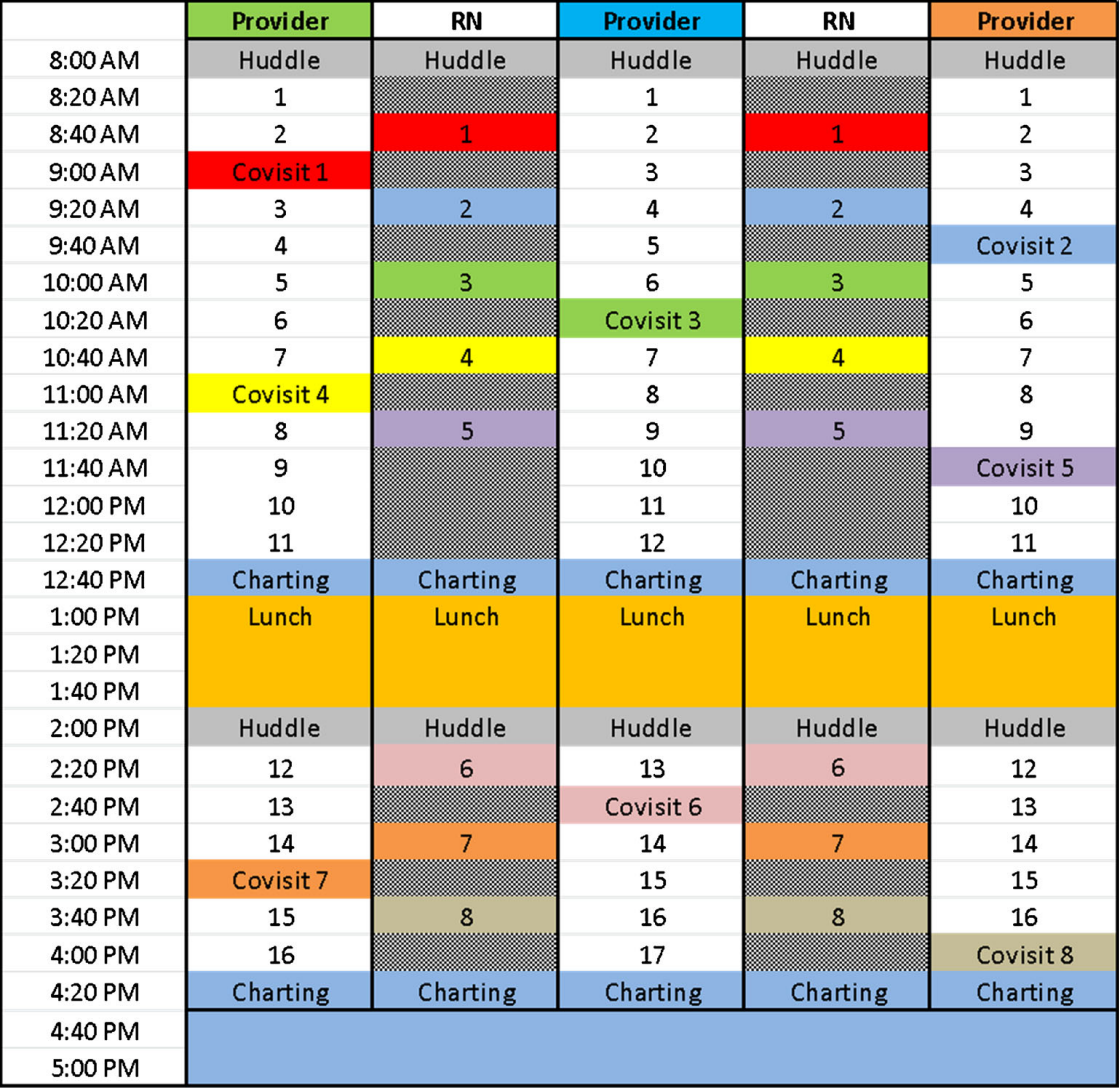
Sample care team schedule with RN co-visits. *Grey* areas represent blocked care slots for co-visit consultation and catch-up charting

Careful preparation was essential for the co-visit model to succeed. We developed a co-visit institute in which nurses, nurse practitioners, and physicians train nurses how to appropriately document co-visits. In addition, nurses received instruction on how to present patients effectively to their provider teammate. Finally, we helped our providers learn how to support the nurse in accomplishing accurate and efficient documentation. The Nursing Services Department developed scheduling guidelines for call center staff and empowered nurses to schedule co-visits for appropriate patients (e.g., those with acute complaints such as respiratory infections) and to designate more complex patient complaints for provider appointments. Similarly, success of the project depended on collaboration with our billing and coding team, electronic health record experts, and compliance officer. We reviewed federal regulations on scribing and state regulations on RN scope to ensure that our approach was compliant.

How did we do? At the first of two pilot sites, daily visit capacity increased by 17 %. At the second site, already a high-productivity facility, double-booked visits were eliminated and capacity grew by 12 %. One provider who was fully booked at 12 patient slots in a morning was able to do three flip visits and bill for 15 patients. Multiple care team members at the pilot sites reported improved satisfaction and work/life balance based on surveys administered at the end of each pilot day. Some providers reported leaving work on time with charting completed for the first time in years.

Nurses loved the new model, affirming that they felt valued and inspired to develop their skills. Patient satisfaction for nurse co-visits averaged 9.5 out of 10, higher than our baseline for provider visits. Since triage was removed from the pod, nurses were able to directly assist patients.

Now, with the added capacity from the co-visit model translating into improved patient access, we anticipate less triage demand. We plan to spread co-visits across our 14 pods in 2015, and will continue to assess whether the extra visits pay for additional staff. With 14 care teams at Clinica, we plan to hire an additional 21 nurses across the organization and an additional five nurses for triage, for a total of 26. Preliminary business case analysis shows that a full year of Pods 2.0 staffing across all care teams, at an average of 2 additional visits per day per medical provider, covers the cost of all additional nursing and medical assistant staff and nets a modest positive revenue.

By transforming Clinica’s RN role through the co-visit model, we are addressing seemingly conflicting goals in primary care—patient access and workforce job satisfaction. By honestly answering the question *“Who is the best person on our care team to help you with your problem today*?,” nurse-led co-visits may be one answer to the complicated issues facing primary care. By having RNs work at the top of their ability, we found a way to improve access without increasing provider stress.

## References

[1] Bodenheimer T (2011). Lessons from the trenches—a high-functioning primary care clinic. N Engl J Med.

[2] Institute of Medicine. The Future of Nursing: Leading change, Advancing Health. October 2010. www.iom.edu/Reports/2010/The-Future-of-Nursing-Leading-Change-Advancing-Health.aspx. Accessed 12 28 2014.

[3] Centers for Medicare & Medicaid Services (CMS) Internet-only Manual IOM 100–02, Chapter 15, Section 60.2.

